# Transcriptome profiling of peanut gynophores revealed global reprogramming of gene expression during early pod development in darkness

**DOI:** 10.1186/1471-2164-14-517

**Published:** 2013-07-29

**Authors:** Han Xia, Chuanzhi Zhao, Lei Hou, Aiqin Li, Shuzhen Zhao, Yuping Bi, Jing An, Yanxiu Zhao, Shubo Wan, Xingjun Wang

**Affiliations:** 1 High-Tech Research Center, Shandong Academy of Agricultural Sciences, Shandong Provincial Key Laboratory of Crop Genetic Improvement, Ecology and Physiology, Jinan 250100, PR China; 2 College of Agriculture, Shandong University, Jinan 250100, PR China; 3 College of Life Sciences, Shandong Normal University, Jinan 250014, PR China

**Keywords:** Peanut (*Arachis hypogaea* L.), Gynophore, High throughput sequencing, Transcriptome, Digital gene expression profiling

## Abstract

**Background:**

After the zygote divides few times, the development of peanut pre-globular embryo and fruit is arrested under white or red light. Embryo development could be resumed in dark condition after gynophore is buried in soil. It is interesting to study the mechanisms of gynophore development and pod formation in peanut.

**Results:**

In this study, transcriptome analysis of peanut gynophore was performed using Illumina HiSeq™ 2000 to understand the mechanisms of geocarpy. More than 13 million short sequences were assembled into 72527 unigenes with average size of 394 bp. A large number of genes that were not identified previously in peanut EST projects were identified in this study, including most genes involved in plant circadian rhythm, intra-cellular transportation, plant spliceosome, eukaryotes basal transcription factors, genes encoding ribosomal proteins, brassinosteriod biosynthesis, light-harvesting chlorophyll protein complex, phenylpropanoid biosynthesis and TCA cycle. RNA-seq based gene expression profiling results showed that before and after gynophore soil penetration, the transcriptional level of a large number of genes changed significantly. Genes encoding key enzymes for hormone metabolism, signaling, photosynthesis, light signaling, cell division and growth, carbon and nitrogen metabolism as well as genes involved in stress responses were high lighted.

**Conclusions:**

Transcriptome analysis of peanut gynophore generated a large number of unigenes which provide useful information for gene cloning and expression study. Digital gene expression study suggested that gynophores experience global changes and reprogram from light to dark grown condition to resume embryo and fruit development.

## Background

Peanut (*Arachis hypogaea* L.) is a world-wide important crop both for oil and protein production. In recent years, great advances have been achieved in peanut functional genomics, proteomics, molecular marker development and other biotechnological based research areas. However, little studies focused on understanding the key biological processes in peanut plants as for instance, the molecular mechanism of peanut geocarpy, which was investigated from the physiological aspect in last century [1-6]. Peanut flowers and finishes pollination above ground as other plants. After fertilization, the activity of an intercalary meristem at the base of the ovary leads to the formation of a gynophore. It carries the ovary and grows in a positive geotropic manner [7]. The zygote cell divides only few times and then both the pre-embryo and pod development are arrested in continuous sun light or regular day/night photo period. When the elongating gynophore pushes the ovary to the soil, the embryo and pod development resumes under dark condition. The penetration of gynophore to soil causes changes in several aspects including light, moisture, nutrition, growth regulator and mechanical stimuli [5]. Light was proven to be the major inhibitor to prevent embryo and pod development. Two studies reported the significant change of phytochrome before and after gynophore soil penetration [2,3]. However, the molecular events downstream of phytochrome signaling remained unknown.

Growth regulators such as auxin, gibberellins, ethylene, ABA and cytokinin play important roles during embryo and fruit development [8-12]. Several studies showed that these growth regulators change either in content or in distribution patterns after gynophore buried in the soil. Shalamovitz reported that IAA content of aerial grown green gynophores, soil grown while gynophores and the young pod (3–8 mm) did not change significantly on the dry weight basis [4]. However, the distribution patterns of IAA before and after gynophore soil penetration showed great difference [6]. The ABA content was significantly decreased after soil penetration and pod development. The content of ethylene in the gynophore after soil penetration was two times higher than in the aerial grown gynophore [4]. The roles of gibberellins in fruit development was evident in *Arabidopsis*, tomato and many other plants [10,11,13]. The content of gibberellins was decreased after the gynophore buried in the soil [7]. However, embryo and pod development was not rescued by exogenous application of plant hormone or hormone inhibitor under light culture condition [4,14]. These results suggested that light mediated arrest of peanut pod development is attributed to an overall change of hormones and other factors. How light regulates hormone biosynthesis and/or signaling and finally leading to the transition of gynophore elongation to pod enlargement is unknown. Brassinosteroid was proven to be key player to interact with light and modulate plant growth and development [15]. Whether this hormone is involved in the regulation of peanut pod development is unknown.

Little advance in understanding the molecular mechanisms of peanut geocarpy was achieved during the past 20 years. Recently, Chen and colleagues analyzed the transcriptome of peanut gynophore and young pod using pyrosequencing and found that the expression levels of two senescence-associated genes were drastically up-regulated in aerial gynophores [16]. The author considered that these two genes may contribute to the embryo abortion of the aerial pod and finally resulted in pod swelling cessation. This exciting study took the first step to understand the mechanism of peanut geocarpic pod development at the molecular level. However, the molecular events bridging light signals and embryo abortion remained to be found.

**Figure 1:**
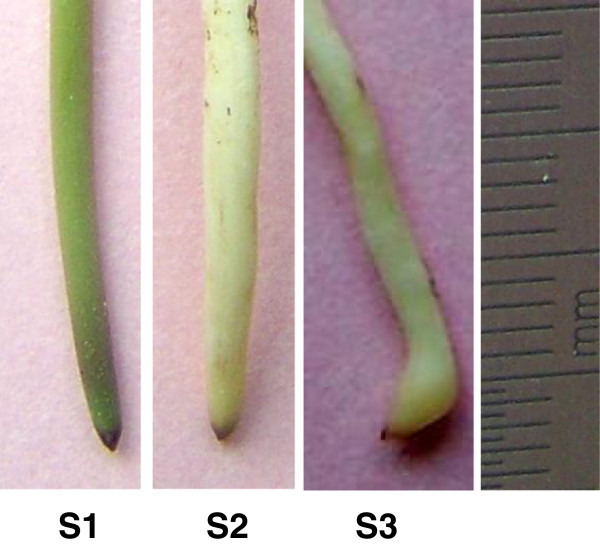
**Three stages of gynophores sampled for experiments.** S1 (stage 1), aerial grown green gynophore; S2 (stage 2), white gynophore after soil penetration without ovary enlargement; S3 (stage 3), gynophore after soil penetration and ovary enlargement.

In this study, we employed RNA-Seq to analyze the transcriptome and profile the overall gene expression of stage 1 (S1) gynophores (aerial grown with green or purple color, 3–5 cm in length), stage 2 (S2) gynophores (The gynophore is buried in the soil for about 3 days. At this time the color of the gynophore is white and the ovary enlargement is not observed) and stage 3 (S3) gynophores (dark-grown gynophores with 2–3 mm small pod) (Figure 1). The RNA-Seq which usually generates much more sequences than pyrosequencing in a single run provides the opportunity to study the key genes that expressed in relatively low levels. Our results suggested a global change in genes expression before and at the beginning of pod initiation. The expression of critical genes and multiple pathways were drastically affected during the transition of gynophore from light to dark conditions. This in turn led to the alteration in hormone biosynthesis and signaling which direct cell growth, division and specification, and finally resumed the embryo development program and start pod enlargement, which was inhibited by light.

## Results

### Transcriptome sequencing and data analysis

A total number of 13293536 reads corresponding to about 120 millions (1196418240) of nucleotides were generated by high throughput sequencing of the gynophore cDNA pool using Illumina HiSeq™ 2000. The raw reads that only have 3′ adaptor fragments were removed before data analysis. Short sequences assembly was performed using SOAPdenovo assembling program to form contigs and scaffolds [17]. More than 353 thousand contigs were assembled, among which the length of the majority contigs (> 84.8%) were less than 200 bp and there are only about 53 thousand contigs are >200 bp in length. By analysis of these contigs, 107364 scaffolds were formed. The length of more than 85% of the scaffolds were ranged from 100–500 bp, while about 14% of scaffolds with a length that longer than 500 bp. We obtained a total number of 72527 unigenes in this study. The average length of unigenes was 394 bp. There is no gap presence within the majority of unigenes (91%) indicating the high quality of sequence assembly (Table 1, Additional file 1: Figure S1). This study generated more unigenes than the total number of peanut unigenes that previously deposited in NCBI database (52468, up to December 2012) (http://www.ncbi.nlm.nih.gov/unigene/?term=arachis). This transcriptome sequences greatly enriched the current peanut sequence database, which could significantly facilitate gene cloning and functional study on the genes involved in peanut growth and development especially in gynophore development.

**Table 1:** Summary of peanut gynophore transcriptome

**Items**	**Value**
Total number of reads	13,293,536
Total nucleotides	1,196,418,240
Average length (bp)	90
Q20 percentage (%)	95.18
GC percentage (%)	44.88
Total number of contigs	353,323
Average length of contigs	150
Total number of scaffolds	107,364
Average length of scaffolds	306
Total number of unigenes	72,527
Average length of unigenes	394

### Annotation of the unigenes

**Figure 2:**
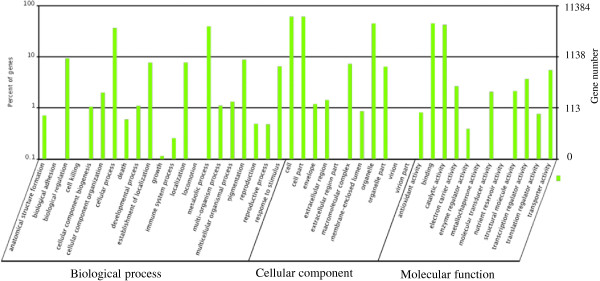
Gene Ontology classification of the identified unigenes.

Annotation of the unigenes was carried out by BLASTX against nr, Swiss-Prot, KEGG and COG (E value <10^-5^) protein database. Information from proteins with the highest similarity to the given unigene was used to annotate the unigene function. Gene Ontology (GO) gene functional classification was performed by Blast2GO program. A total number of 47044 unigenes could be annotated by GO classification system. Based on the GO annotation the unigenes were classified into 44 different groups belonging to three main categories: biological process, cellular component and molecular function (Figure 2). The genes involved in cellular process and metabolic process were dominant in the “Biological process” category. “Cell”, “Cell part” and the “Organelle” are the top three abundant categories in “Cellular com-ponent”. While “Binding” and “catalytic activity” are dominant in the “Molecular function” categories (Figure 2).

**Figure 3:**
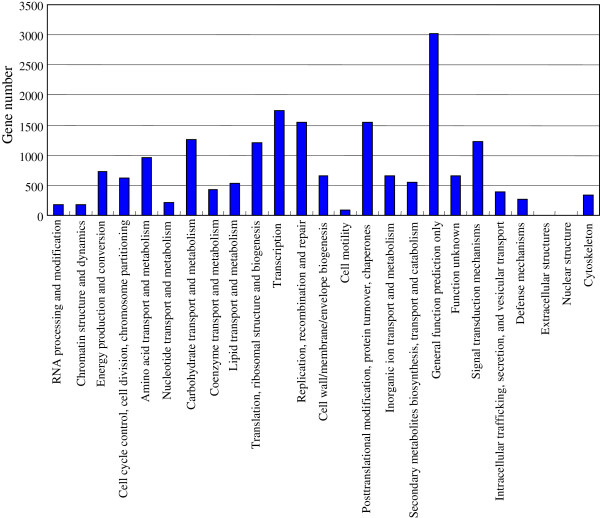
COG classification of the identified unigenes.

Furthermore, COG (Clusters of Orthologous Groups) classification system was also used for function prediction and classification. The results showed that 19000 unigenes could be annotated through COG system. Among these genes 3020 (15.89%) unigenes that predicted to have “General function” represented the most abundant group. There were more than 1500 unigenes under each of the following categories: “Transcription”, “Replication” and “Posttranslational modification” (Figure 3). Unigenes identified in this study were predicted to be involved in 115 metabolic pathways base on the comparison of these genes with the KEGG database.

Unigenes generated from the transcriptome sequencing were analyzed by BLAST for CDS prediction. Out of the 72527 unigenes, CDSs of 43660 unigenes could be predicted. The rest of unigenes whose CDS were not identified by BLAST were subjected to further analyze using ESTscan for CDS prediction and 4095 CDSs were predicted. Together, CDS of more than 65% of the unigenes could be predicted. The majority of all CDSs predicted in this study (>90%) have no GAP within the sequence. The length distribution and GAP distribution were shown in Additional file 2: Figure S2.

Most genes involved in plant circadian rhythm, intra-cellular transportation, plant spliceosome, eukaryote basal transcription complex, genes encoding ribosomal proteins, brassinosteriod biosynthesis, light-harvesting chlorophyll protein complex, phenylpropanoid biosynthesis and TCA cycle are identified in this study (Additional file 3: Figure S3-S12). The expression of specific peanut phytochrome genes was detected. Few unigenes encoding phytochrome A and B were detected hundreds of times. A phototropin and bacterial phytochrome were detected at low abundance. It is interesting to find an enrichment of the genes encoding enzymes involved in the brassinosteriod biosynthetic pathway to compare with other plant hormone biosynthetic pathways such as gibberellins and auxin. The roles of brassinosteriod in peanut gynophore and pod development have never been investigated before. Further studies are necessary to understand the function of this hormone in peanut pod development. Similarly, phenylpropanoid biosynthesis related genes were also highly enriched in the sequences identified in this study. The transcriptome data is available from GenBank (BioProject: PRJNA181974, SRA: SRR827601, Unigene number: GAIG00000000).

### Digital gene expression (DGE) profiling

Three DGE libraries (S1, S2 and S3) were constructed using gynophores with different developmental stages as described under method section. By sequencing S1: 11,743,278; S2: 11,844,796; and S3: 12,348,662 reads were generated, respectively. After removal of reads that containing unreadable nucleotides, reads containing only the adaptor sequences and reads with low quality, we obtained the clean reads for further analysis. Totally, 11.67, 11.78 and 12.27 millions of clean reads from S1, S2 and S3 library was obtained, respectively. This sequencing depth was saturated for gene identification (Additional file 4: Figure S13). Gene coverage is the percentage of a gene covered by reads. The result showed that the majority of genes (70%) in the three samples showed > 50% coverage by the reads (Additional file 5: Figure S14). The gene expression level was then calculated by using RPKM method (Reads Per kb Per Million reads) to eliminate the influence of different gene length and sequencing discrepancy on the calculation of gene expression.

### Differentially expressed genes in S1 and S2

When gene expression levels were compared between S1 and S2, the expression of a large number of genes was found to be up- or down-regulated (RPKM ≥2 folds). Specifically, the expression of 2049 genes was up-regulated while 2407 genes were down-regulated in S2 to compare with S1 (Additional file 6: Table S1). A large number of these genes encode proteins with unknown/hypothetical function or genes with no hit in the database. These groups of proteins accounted about 60% (1224/2049) and 65% (1554/2407) of the total up- and down-regulated genes, respectively.

More than sixty unigenes annotated as transcription factors including WRKY, MYB, B3, AP2-EREBP, NAC, MADS-box, GRAS, CCAAT-binding, C3HL domain class, bZIP, basic leucine zipper and ethylene-responsive transcription factors were found up-regulated in S2. The transcript abundance of some transcription factors was about 10 times higher in S2 than in S1, for example, MYB92 and R2R3-myb transcription factor. The transcripts of CBF/DREB transcription factor and two AP2-EREBP transcription factors could be detected only in S2. The expression of four AP2 encoding unigenes showed decreased level in S2.

Unigenes encoding proteins involved in several hormone metabolism and signal transduction pathways were found differentially expressed in S1 and S2. Tryptophan is the substrate of tryptophan dependant IAA biosynthesis pathway. The expression of tryptophan synthase beta subunit gene was only detected in S2. One to two unigenes encoding auxin-repressed protein, auxin-induced protein, auxin response factor 4, auxin efflux carrier family protein and auxin conjugate hydrolase were found to up-regulated after gynorphore soil penetration. However, nine unigenes encoding auxin-induced proteins were found down regulated in S2 which was in agreement with the lowered auxin content in this stage. Six unigenes encoding auxin influx and efflux carrier protein were found down regulated in S2 indicating that after soil penetration auxin polar transport was altered. Six unigenes annotated as GA receptor or GID1 like were found up-regulated, while one GID1 gene was down regulated in S2. GA_2_ oxidase is a major enzyme catalyzing the inactivation of biological GA. The expression of GA_2_ oxidase gene was higher in S2 than in S1. Although one GA_20_ oxidase gene was detected to up-regulated in S2, the transcripts abundance of this gene was low. The expression of two other GA_20_ oxidase genes with much higher expression level (30–100 folds) was down regulated in S2 indicating the major trend of GA biosynthesis was negatively regulated in dark grown gynophores. The ent-kaurenoic acid oxidase which catalyzes early stage of GA biosynthesis was also down regulated upon soil penetration. These results indicated a decreased GA content in dark grown gynophore. Low GA content could result in the accumulation of DELLA protein, the major repressor of GA signaling pathway. However, the expression level of DELLA unigenes was decreased in S2 (Additional file 6: Table S1).

In S2, the expression of brassinosteroid receptor and BRASSINOSTEROID INSENSITIVE 1-associated receptor kinase 1 genes was down-regulated. The changing pattern of ethylene related genes varied. Three unigenes encoding 1-aminocyclo- propanecarboxylic acid oxidase (ACO), a key enzyme in ethylene biosynthesis, were found significantly up-regulated in S2. Genes involved in flavonoid biosynthesis were up-regulated. However, the expression of many enzymes catalyzing the later steps of flavonoid biosynthesis pathway was decreased in S2 which was agree with the decreased pigmentation of the dark grown gynophore. The decreased CHS expression may provide more substrate for lignin biosynthesis, which is a major event during peanut pod development.

More than eighty unigenes encoding proteins involved in light signal transduction and photosynthesis related processes were identified in digital expression data set. Reasonably, the expression of all these genes were found significantly decreased after gynophore soil penetration. The expression level of many of these genes decreased more than 10 times in S2, for examples, genes encoding phototropin, phytochrome kinase substrate 1-like protein, thylakoid lumenal 29.8 kDa protein, ribulose-,5-bisphosphate carboxylase, early light induced protein, photosystem I reaction centre and chlorophyll a/b-binding protein. Several phytochrome A and B were detected in the gene profiling study. However, their expression levels were not significantly different between S1 and S2.

The ubiquitin ligase COP1 is a key negative regulator of plant photomophogenesis. In dark conditions, COP1 is localized in nucleus and mediate the repression of light regulated genes. Upon light irradiation, COP1 is re-localized mainly in the cytoplasm and release the repression of its target genes. The expression of four unigenes encoding COP1 was drastically decreased in dark-grown gynophores. Previous studies indicated that the transcriptional expression of CIP7 (COP1 interacting protein 7) was up-regulated by light [18]. The expression of two unigenes encoding CIP7 was drastically decreased in dark-grown gynophores. Two unigenes annotated as root phototropism protein (RPT) were found down regulated in S2 to compare with S1. RPT2 is a signal transducer of phototropism response in *Arabidopsis* and could modulate phototropin 1 which showed a decreased expression in S2. Red/far-red and blue light could induce RPT2 expression which plays important roles in hypocotyl phototropism. RPT2 together with phototropin 1 modulate auxin gradient in phototropism response [19]. Lipoxygenase is a group of enzymes that catalyze the dioxygenation of polyunsaturated fatty acid. The products of the reaction may play important roles in signal transduction and disease resistance. Twelve unigenes encoding lipoxygenase were found up-regulated in S2. Together, these data indicated that gynophores experience global changes and reprogram from light-to dark-grown condition to resume embryo and fruit development.

### Differentially expressed genes in S1 and S3

The expression of 8398 genes was up-regulated while 6536 genes were down-regulated in S3 to compare with S1 (Additional file 7: Table S2). About 70% (10439) of these genes were annotated as unknown protein, hypothetical protein or with no hit in the data base. Genes involved in auxin, gibberellins and ethylene biosynthesis and signaling transduction as well as the light signal and photosynthesis related genes showed changed expression patterns.

### Up-regulated genes in S3

Interestingly, several genes encoding key components of light signaling pathways were found up-regulated in S3 to compare with S1, for example, phytochrome A, phytochrome B, phototropin, cryptochrome 1 and cryptochrome 2. However, the expression of *PhyA*, *PhyB*, *Cry1* and *Cry2* did not display significant difference between S1 and S2. And the expression of phototropin genes was down-regulated in S2.

More than 150 transcription factor encoding genes were up-regulated in S3: MYB transcription factor, bZIP transcription factor, WRKY transcription factor, several MADS-box transcription factors, heat shock transcription factors, AP2 domain-containing transcription factor and GRAS family transcription factors, three ARF (auxin responsive factor) domain class transcription factors and BEL1-like transcription factor genes. Squamosa promoter-binding protein was also found up-regulated in S3. BEL1-like transcription factor could interact with KNOTTED I to modulate different development process in plants. Agamous-like 1 is a key regulator in fruit growth and ripening [9]. The expression of two genes encoding agamous-like 1 protein increased more than 20 folds in S3. Totally, 30 unigenes encoding heat shock protein transcription factor were detected in the transcriptome analysis. In S3 stage, 6 of them were detected 2 fold up-regulated. Out of these genes, only 2 were detected to be down-regulated in S3.

The expression pattern of lipoxygenases genes in S3 clearly differed from that in S2. The expression level of nine abundantly expressed (up to 15 thousand reads were detected in S1) lipoxygenase genes showed highest expression level (except one gene showed highest expression in S2) in S1 and decreased in S2 and S3. While 16 unigenes encoding lipoxygenase showed more than 2 folds up-regulated in S3 to compare with S1. The transcript abundance of these up-regulated genes was generally low from few reads to few hundred reads. Many unigenes encoding plant hormone biosynthesis and signaling pathway component including the auxin, gibberellin, ethylene, brassinosteroid and ABA were detected up-regulated in S3. However, many other genes involved in plant hormone biosynthesis and signal transduction pathways showed different expression patterns between S1 and S3 as described below.

### Down regulated genes in S3

Phytochrome kinase substrate 1-like protein, phototropin, photosystem II type I chlorophyll a/b-binding protein, photosystem I reaction centre, photosystem I psaH protein and an early light induced protein were all down-regulated in S3. Phytochrome kinase substrate 1-like protein together with phototropin play critical role in root phototropism and gravitropism. Multiple unigenes encoding phototropin and phytochrome kinase substrate 1-like protein were down-regulated in S2 and in a larger extent in S3 suggesting the importance of these genes during the transition from gynophore elongation to pod initiation. GIGANTEA, a gene involved in circadian clock and phytochrome signaling, was down-regulated in S3. It is understandable that the light signal and photosynthesis related genes showed decreased expression in S3 in the dark condition.

The expression of eight unigenes encoding condensins, members of SMC (Structural Maintenance of Chromosomes) family, decreased in S3 indicating the altered cell division and differentiation. Cellulose is the major component of plant cell wall and cellulose synthase catalyse the formation of cellulose. Thirty nine unigenes annotated as cellulose synthase were found down regulated 2–30 folds in S3 suggesting the significantly reduced synthesis of cellulose during this stage. Six unigene encoding chitinase like proteins showed decreased expression in S3. The expression of several expansins, a group of proteins considered to induce cell wall extension in acidic condition [20], was decreased in S3. Multiple unigenes that encoding tubulin, extensin/nodulin protein, actin-depolymerizing factor, and cyclin showed decreased expression in S3. UDP-glucose pyrophosphorylase which is important in cell wall synthesis was found to be decreased significantly in S3. Together, these results indicated that cell growth and division experience drastic changes in the dark condition and may be closely associated with the change of geotropic growth and meristem activity and eventually led to the horizontal orientated pod initiation.

Among the down-regulated transcription factors in S3, the bZIP, MYB, AP1, AP2 and dof transcription factors represented the dominant families. The expression of two GATA encoding genes showed decreased expression in S3; however, there was one GATA transcription factor showed increased expression in S3. More than 100 unigenes encoding heat shock protein or heat shock protein transcription factors were detected in the transcriptome analysis. In S3, five heat shock protein transcription factors were detected up-regulated. A large number of transporter genes exhibited decreased expression in S3. These transporters included ABC transporter family, carbohydrate transporters, sulfate transporter, sodium/calcium exchanger family, vesicular transport related proteins, potassium channel, oligopeptide transporter, nucleobase-ascorbate transporter, nitrate transporter, amino acid transporter, lipid transfer protein, ERD6-like transporter, copper transport protein, cation-chloride cotransporter, Ca2+/H + −exchanging protein, boron transporter, anion exchanger family protein and acyl carrier protein.

Many unigenes encoding proteins involved in hormone biosynthesis, transportation and signal transduction were detected to be down-regulated in S3. For example, four unigenes encoding GA_20_ oxidase, two ent-kaurenoic acid oxidase encoding genes and the GID1 encoding gene were down-regulated. Eleven unigenes encoding auxin efflux and influx carriers, 21 auxin induced protein genes, several GH3 family protein genes and indole-3-acetic acid-amido synthetase gene were down-regulated. Interestingly, the expression of six unigenes involved in brassinosteroid biosynthesis and signaling was also found to be decreased in S3 similarly to several genes involved in ethylene response. The expression of phytosulfokine precursor gene could not be detected in S3, while its transcripts were present in S1 and S2 with a low abundance. Together, altered expression of transcription factor genes and genes involved in plant hormones were detected, indicating the substantial changes in growth and development of gynophore from light-grown to dark-grown condition.

### qRT-PCR validation of DGE results

The transcription levels of 27 genes were determined by qRT-PCR to valid our DGE results. The primer pairs used for qRT-PCR were designed based on nucleotide sequences of the transcriptome result (Additional file 8: Table S3). Actin gene was chosen as internal control. Additional file 9: Table S4 showed the expression changes of these genes in S1, S2 and S3 gynophores. Twenty four genes showed the similar expression patterns with DGE results (Additional file 9: Table S4). However, the expression of three genes displayed different patterns between qRT-PCR and DGE results (Additional file 9: Table S4). For example, qRT-PCR showed that the expression of unigene16478 increased in S2 to compare with S1, while the DGE result showed that this unigene was down-regulated in S2. Previous study observed this discrepancy when studying the gene expression of *Camellia sinensis* using these two methods. The tag-based nature of DGE analysis may cause inaccuracy estimation of gene expression [21].

## Discussion

Light repressed peanut embryo and pod development could be reactivated in darkness. The downstream alteration at molecular level following the light to dark transition was largely unknown. The transcriptome and digital gene expression results showed that the expression of genes in many biosynthetic pathways and signal transduction pathways were changed when gynophore grown from the light to dark condition.

Several genes involved in light signaling transduction showed different regulation in S1, S2 and S3. Post translational regulation is crucial for phytochrome and cryptochrome regulation; however, we can not exclude their possible roles in peanut pod development at transcription level. Phototropin and cryptochrome are photoreceptors in blue light and UV-A light signal transduction pathways [22,23]. The changed expression patterns of these genes reflected the involvement of blue light and UV light signaling besides red and far-red light signaling [3] in gynophore to pod development transition.

Phytochrome kinase substrate 1 (PKS1) is an important phytochrome signaling component and could be phosphorylated by phytochrome [24]. It may represent an integrator between phototropin mediated blue light signaling and phytochrome mediated signaling transduction pathways [25]. Previous study showed that light could transiently increase PKS1 mRNA level in the elongation region of hypocotyl and root under very low influence response mode, while prolonged incubation in far-red light could positively promoted the accumulation of its RNA and protein [24]. Interestingly, we found two unigenes encoding PKS1 like protein in S2 and three PKS1 like protein in S3 were significantly down-regulated. A key gene that play important roles in plant light signal transduction and controlling of flowering time, CONSTANS, was up- regulated in S3. The expression of CONSTANS gene was controlled by the circadian clock, and its mRNA abundance rises about 12 hours after dawn and stays high through the night. However, the COSTANS protein was targeted for degradation by the proteasome in darkness. Therefore, the protein level of CONSTANS was unknown in S3 gynophore which buried in darkness for about 9 days. The expression of COP1 and COP1 interacting protein, key components of light signaling [26,27], varied in these three stages.

Based on GO classification 171 unigenes (~3.8% of the 2 x changed unigenes) were detected to response to light stimuli or response to radiation. Eighty of these genes were annotated as unknown protein, predicted or hypothetical protein indicating that many important genes involved in gynophore growth transition from light to dark were uncharacterized. The differential expression patterns of these components of light signal transduction pathways suggested the complex regulation in these three developmental stages of gynophore.

Early studies demonstrated that auxin, gibberellins, ABA and ethylene were affected differentially before and after soil penetration of peanut gynophore. Plant hormones could be the down stream target of light response. Many unigenes encoding auxin repressed protein were expressed at a low level in S1. However, their expression increased steadily after soil penetration and reached more than 2 and 6 folds at S2 and S3, respectively. This result was consistent with the result obtained from black locust [28]. The gene expression data was in agreement with the decreased IAA content (data not shown) in S2 and S3. *AUX1* encodes a membrane protein involved in auxin uptake is a key factor for lateral root development and gravitropism in *Arabidopsis*[29,30]. AUX1 like 3 unigenes were down-regulated in S2 and S3. *Arabidopsis* Aux/IAA proteins repressed transcription of auxin-responsive reporter genes. The expression of one peanut AUX/IAA protein gene decreased in S2 to compare with S1. Two unigenes encoding AUX/IAA protein decreased drastically (4–34 folds) in S3 to compare with S1. These results were coincided with the loss of gravitropism after gynophore soil penetration. Together, our results indicated that auxin biosynthesis, transportation and signaling may represent key downstream effectors of light signaling during gynophore growth and pod initiation. In addition, genes in gibberellins, ABA and ethylene biosynthesis or signaling were detected to up-or down-regulated in these three stages (Additional file 6: Table S1, Additional file 7: Table S2).

In recent years, extensive studies have revealed the key regulatory roles of brassinosteroid in plant growth and development. Brassinosteroid could be perceived by BRI1 (BRASSINOSTEROID INSENSITIVE 1) and then activates BZR family of transcription factor which could physically interact with PIFs (phytochrome interacting factors) and DELLA protein [15]. Therefore, change in biosynthesis or signaling of BR would not only affect endogenous hormone level and/or hormone signalling but would also affect light signals and responses. However, there is no report about BR content or signal transduction on peanut pod development. Interestingly, we found in S2 the decreased expression of one brassinosteroid receptor and three BRASSINOSTEROID INSENSITIVE 1-associated receptor kinase unigenes. In S3, beside three BRI1-associated receptor kinase 1 unigenes, one brassinosteroid LRR receptor kinase unigene and two brassinosteroid biosynthetic protein unigenes were down-regulated. Two brassinosteroid receptor unigenes were up-regulated in S3. These altered expression patterns of BR biosynthesis and signal transduction related genes suggested the involvement of brassinosteroid in gynophore elongation and pod enlargement initiation.

Upon changes in light signaling, changes in hormone biosynthesis/signaling, down stream alteration related to cell division and cell wall formation were observed in this study. Cellulose is the major component of plant cell wall and cellulose synthase catalyse the formation of cellulose. Mutation of cellulose synthase gene caused decrease in cellulose content and resulted in cell swelling; restrict root and hypocotyl elongation [31,32]. Thirty eight unigenes encoding cellulose synthase were down-regulated in S3 compared to S1, indicating a drastic change in synthesis of cell wall components under dark condition. Other genes involved in cell division, cell cycle and cell components are significantly enriched among unigenes with >2 folds changes in S3 (Additional file 7: Table S2). Chitinase-like proteins are important for cellulose biosynthesis and mutation of their encoding genes resulted in root cell swelling and over production of ethylene [33,34]. Six unigenes encoding chitinase like proteins showed decreased expression in S3.

Condensin complexes play critical roles in organizing chromosome structure and facilitate chromosome segregation during cell division. The mutation of SMC, key component of condensing complex, resulted in defect in embryo and endosperm development [35]. Antisense repression of SMC expression resulted pleotropic post-embryonic developmental defects such as primary shoot meristerm organization, root growth retardation which may be caused by abnormal cell division and differentiation [36]. The expression of eight unigenes encoding condensin decreased in S3.

Extensive studies demonstrated that heat shock proteins play key roles in plant stress response, including high temperature, drought, salinity and cold [37,38]. Heat shock proteins are crucial in evolutionary aspects as well [38,39]. The cost while plants expressing these proteins to adapt the stressful environment was observed. Constitutive expression or over expression of heat shock proteins led to abnormal growth and development including reduced fertility and development rate [38]. When gene encoding Hsp101 was mutated in maize, the primary root growth of the mutant seedlings was greatly promoted [40]. Mutation of Heat Shock Response Binding Protein (HSBP), a negative factor of heat shock response, led to embryo abortion in *Arabidopsis* and Maize [41,42]. We identified more than 100 heat shock related unigenes in gynophore transcriptome. Interestingly, more than forty unigenes encoding heat shock proteins or heat shock transcription factors were found up-/down-regulated in S2 and S3. Among which the expression level of three highly expressed Hsp70 unigenes decreased drastically in S3. Is the accumulation of these proteins in aerial grown gynophore associated with peanut embryo development arrest just like what happened in maize and *Arabidopsis*? Is the accumulating of heat shock proteins reflecting the evolutionary protective mechanism considering the harsh environment of peanut origin area? It is probably interesting to investigate whether heat shock proteins are involved in the evolution of peanut geocarpy, an effective way to minimize development damage under extreme stress environment.

## Conclusions

Transcriptome analysis of peanut gynophore generated > 70 thousand unigenes which provide useful information for gene cloning and expression study. These unigenes were used as reference sequences for our digital gene expression study of peanut gynophore. We found the alteration in plant hormone biosynthesis and signaling, and other key pathways after gynophore soil penetration. The expression of many key genes differed significantly in three developmental stages of gynophore. It is a global change and reprogramming rather than modification of a particular pathway to release the light inhibition and resume embryo and fruit development in darkness.

## Methods

### Plant material

The cultivated peanut Luhua-14 was used in this experiment. The plants were grown in the experimental farm of Shandong Academy of Agricultural Sciences. The downward growing gynorphore (with green or purple color) which are 3–5 cm in length was assigned as stage 1 (S1). The stage 2 (S2) gynophores were those that buried in the soil for about three days with thicker diameter than S1 gynophores. The color of S2 gynophores was white and enlargement of the ovary region was not observed. Stage 3 (S3) gynophores were those that buried in soil for about nine days. The ovary region of S3 gynophores was obviously enlarged although in a small size (Figure 1). About 1 cm from top of S1, S2 and S3 gynorphores were cut from the plants separately and immediately frozen in liquid nitrogen for RNA extraction.

### RNA extraction, cDNA synthesis and high throughput sequencing

Total RNA was isolated from the frozen gynophore by using Trizol Reagent (TaKaRa, Inc., Dalian, China) according to the manufacturer’s instructions. The quality and quantity of the purified RNA from each sample was determined by Agilent 2100. Beads with Oligo(dT) were used to enrich polyA mRNA. Equal amount of polyA mRNA from S1, S2 and S3 gynophores were pooled together. Fragmentation buffer was added to interrupt mRNA to short fragment (200–700 nt). These short mRNA fragments were used as templates to synthesize the first strand cDNA. The second strand cDNA was synthesized using DNA polymerase I (New England Biolabs), RNase H (Invitrogen), dNTPs and buffer. After purified by QiaQuick PCR kit and washed with EB buffer for end repair, polyA tails and adaptors were added to the fragments. Fragments with suitable size were recovered from the gel and amplified by PCR. The PCR products were sequenced using Illumina HiSeq™ 2000.

### Data analysis

After sequencing, raw reads were acquired, and then obtained the clean reads by removing the sequences contain only adapters. The clean reads were used for denovo assembly which is carried out with the program SOAPdenovo. Firstly, the clean reads with certain length of overlap were combined to form contigs. Next, the clean reads were used to re-map to contigs, and then scaffolds were made. Finally, pair-end reads were used again for gap filling to generate unigenes. The functional annotation, GO functional analysis and KEGG pathway analysis of these unigenes were carried out by BlastX (E value < 0.00001) against Nr, Swiss-Prot, KEGG and COG Database. The coding region and sequence direction was determined according the annotation results and the software ESTScan. Whole dataset of our transcriptome has been deposited in GenBank database (BioProject: PRJNA181974, SRA: SRR827601, Unigene number: GAIG00000000).

### Transcriptome and digital gene expression profile analysis

High quality mRNA was isolated from three samples (S1, S2 and S3) and interrupted to short fragments (about 200 nt), then the three samples were sequenced separately. After filtering adaptor fragments and low quality read, clean reads were acquired. Then the software SOAPaligner/soap2 were used to map the clean reads to reference sequences according to the criteria that no more than two bases mismatches were allowed in the alignment. The reference sequences were composed of unigenes identified in the current transcriptome study and unigenes available in GenBank (http://www.ncbi.nlm.nih.gov/unigene?term=arachis), as well as peanut ESTs in GenBank(http://www.ncbi.nlm.nih.gov/nucest/?term=arachis). A series of statistical and bioinformatical analysis were followed. Statistical analysis was conducted to summarize the number of clean reads that align to the reference genes, which providing us the general information of the project. Sequencing saturation analysis was used to access the saturation of sequencing data of each sample. When the sequencing data was saturated, the number of genes mapped by clean reads tends to be stabilized. The randomness of the sequencing was evaluated by analysis the distribution of reads on reference sequences. The gene expression level is calculated by the numbers of reads mapped to the reference sequences, and then normalized to RPKM (Reads Per kb per Million Reads) with the following formula: PPKM = [10^6^C/(NL/10^3^)]. Given RPKM (A) to be the expression of gene A, C is the number of reads that uniquely aligned to the gene A. N is the total number of reads that uniquely aligned to all genes. L is the number of bases of gene A. After screening of differentially expressed genes (DEGs), GO function analysis and KEGG pathway analysis were carried out.

### qRT-PCR validation of DGE results

To verify the results of the digital gene expression profiling genes were randomly selected for validation by quantitative real-time PCR (qRT-PCR). The gene-specific primers used for qRT-PCR are listed in Additional file 8: Table S3. Total RNA was isolated from gynophore by using Trizol agent (TaKaRa, Dalian, China) according to the manufacturer’s instructions. After DNase I-treatment, the first-strand cDNA was synthesized with an oligo(dT) primer using a PrimeScript™ first-strand cDNA synthesis kit (D6110A; TaKaRa, Dalian, China). The real-time PCR was performed using FastStart Universal SYBR Green Master (Roche, USA). Each 20 μl reaction mixture contained 10 μl FastStart Universal SYBR Green Master, 0.5 μl of 10 μmol/L gene-specific primers, and 1 μl of 50-fold diluted first-strand cDNA. The PCR reactions were run in an ABI PRISM 7900HT sequence detection system (Applied Biosystems) using the following programme: 95°C for 10 min, then 40 cycles of 95°C for 15 s and 60°C for 1 min. Peanut *Actin* was used as the reference gene to normalize the gene expression level. PCR products were verified by melting curve analysis, by which non-specific products can be detected. Quantification of the relative changes in gene expression was performed using the 2^-△△CT^ method as described [43].

## Competing interests

The authors declare that they have no competing interests.

## Authors’ contributions

XW, SW and YZ designed the experiment and drafted the manuscript. HX and CZ carried out most of the experiment, analyzed the transcriptome and digital gene expression data, wrote the materials and method part of the manuscript. LH and SZ carried out qRT-PCR experiment. YB, AL and JA grew the plants and prepared the samples. All authors read and approved the final manuscript.

## Supplementary Material

Additional file 1: Figure S1 Length distribution of contigs (A), scaffolds (B) and unigenes (C), GAP of the unigenes (D). Click here for file

Additional file 2: Figure S2 Length and GAP distribution of CDS predicted by BLAST (A, B) and ESTscan (C, D) program. Click here for file

Additional file 3: Figure S3-S12 Genes identified in peanut transcriptome and the related pathways. Click here for file

Additional file 4: Figure S13 Sequencing depth was saturated for gene identification. S1 (stage 1), aerial grown green gynophore; S2 (stage 2), white gynophore after soil penetration without ovary enlargement; S3 (stage 3), gynophore after soil penetration and ovary enlargement. Click here for file

Additional file 5: Figure S14 Gene coverage analysis of S1, S2 and S3. S1 (stage 1), aerial grown green gynophore; S2 (stage 2), white gynophore after soil penetration without ovary enlargement; S3 (stage 3), gynophore after soil penetration and ovary enlargement. Click here for file

Additional file 6: Table S1 Differentially expressed genes in S1 and S2. S1 (stage 1), aerial grown green gynophore; S2 (stage 2), white gynophore after soil penetration without ovary enlargement. Click here for file

Additional file 7: Table S2 Differentially expressed genes in S1 and S3. S1 (stage 1), aerial grown green gynophore; S3 (stage 3), gynophore after soil penetration and ovary enlargement. Click here for file

Additional file 8: Table S3 Gene-specific primers used in quantitative real-time PCR. Click here for file

Additional file 9: Table S4 Verification of DGE results by qRT-PCR. The results showed the expression changes of 27 randomly selected genes. Three biological replicates, R1, R2 and R3 were used in this study. Average represent the mean of expression folds of S2/S1 or S3/S1; SD represent the standard derivation of the mean (n = 3); Seq Result denotes the DGE expression level. Click here for file
