# Supplementary material for: Transcriptome profiling of peanut gynophores revealed global reprogramming of gene expression during early pod development in darkness
Source: BMC Genomics. 2013 Jul 29;14:517. doi: 10.1186/1471-2164-14-517 (PMC3765196; doi:10.1186/1471-2164-14-517)
Supplement: Additional file 1: Figure S1 — Length distribution of contigs (A), scaffolds (B) and unigenes (C), GAP of the unigenes (D). [file 1471-2164-14-517-S1.ppt]

## Slide 1
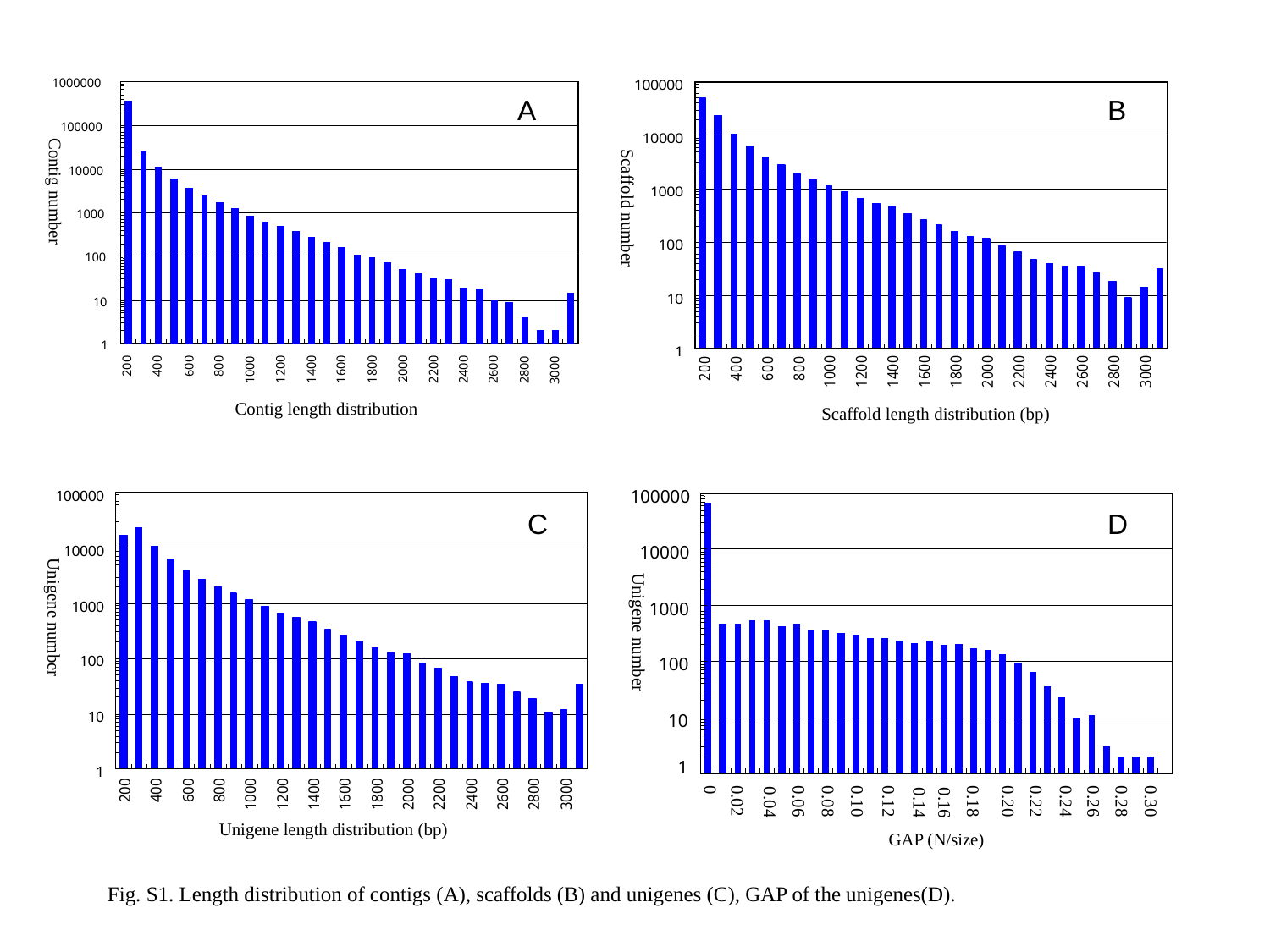

B
Scaffold number
Scaffold length distribution (bp)
1000000
100000
10000
1000
100
10
1
200
400
600
800
2000
1200
1400
1600
1800
2200
2400
2600
1000
2800
3000
A
Contig number
Contig length distribution
C
Unigene number
Unigene length distribution (bp)
100000
D
10000
Unigene number
1000
100
10
1
0.02
0
0.06
0.08
0.10
0.12
0.18
0.20
0.22
0.24
0.26
0.28
0.30
0.04
0.14
0.16
GAP (N/size)
Fig. S1. Length distribution of contigs (A), scaffolds (B) and unigenes (C), GAP of the unigenes(D).
