# Supplementary material for: Transcriptome profiling of peanut gynophores revealed global reprogramming of gene expression during early pod development in darkness
Source: BMC Genomics. 2013 Jul 29;14:517. doi: 10.1186/1471-2164-14-517 (PMC3765196; doi:10.1186/1471-2164-14-517)
Supplement: Additional file 2: Figure S2 — Length and GAP distribution of CDS predicted by BLAST (A, B) and ESTscan (C, D) program. [file 1471-2164-14-517-S2.ppt]

## Slide 1
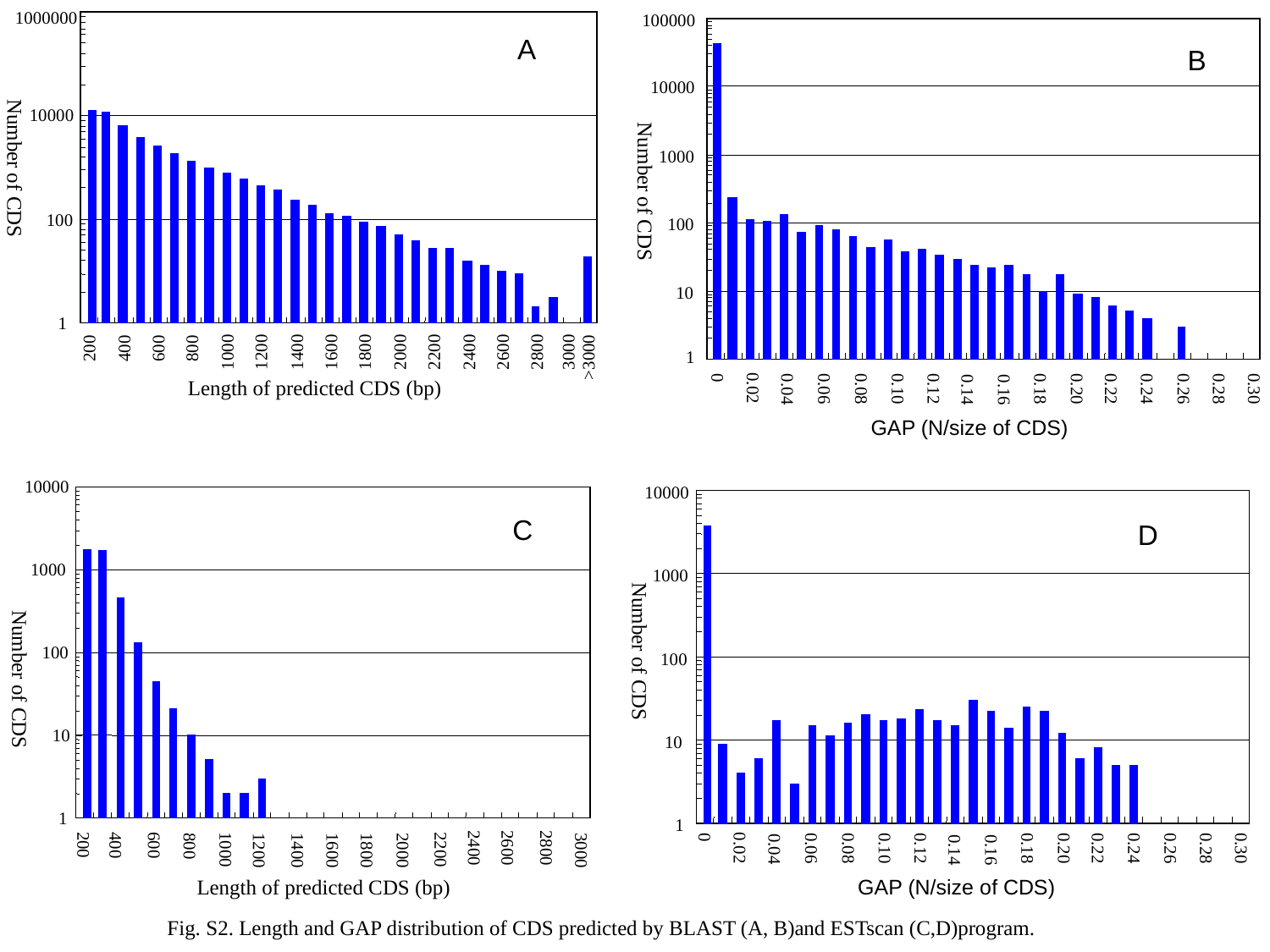

1000000
Number of CDS
10000
100
1
200
400
600
800
1000
1200
1400
1600
1800
2000
2200
2400
2600
2800
3000
>3000
Length of predicted CDS (bp)
A
100000
B
10000
Number of CDS
1000
100
10
1
0.02
0
0.06
0.08
0.10
0.12
0.18
0.20
0.22
0.24
0.26
0.28
0.30
0.04
0.14
0.16
GAP (N/size of CDS)
10000
1000
Number of CDS
100
10
1
2400
2600
2800
200
400
600
1000
2200
2000
800
1200
1400
1600
1800
3000
Length of predicted CDS (bp)
C
10000
D
1000
100
10
1
0.02
0
0.06
0.08
0.10
0.12
0.18
0.20
0.22
0.24
0.26
0.28
0.30
0.04
0.14
0.16
GAP (N/size of CDS)
Number of CDS
Fig. S2. Length and GAP distribution of CDS predicted by BLAST (A, B)and ESTscan (C,D)program.
