# Supplementary material for: Transcriptome profiling of peanut gynophores revealed global reprogramming of gene expression during early pod development in darkness
Source: BMC Genomics. 2013 Jul 29;14:517. doi: 10.1186/1471-2164-14-517 (PMC3765196; doi:10.1186/1471-2164-14-517)
Supplement: Additional file 3: Figure S3-S12 — Genes identified in peanut transcriptome and the related pathways. [file 1471-2164-14-517-S3.ppt]

## Slide 1
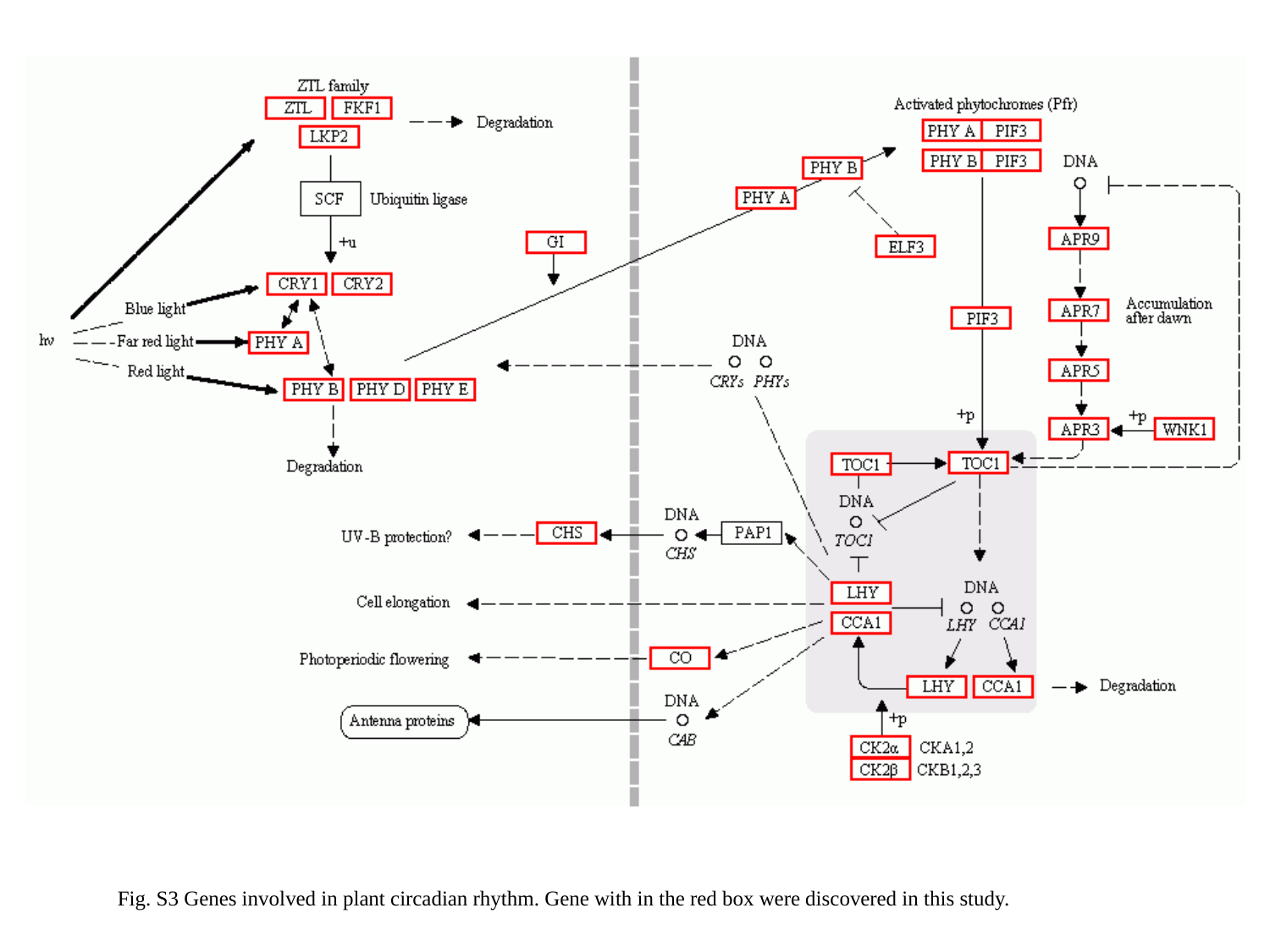

Fig. S3 Genes involved in plant circadian rhythm. Gene with in the red box were discovered in this study.

## Slide 2
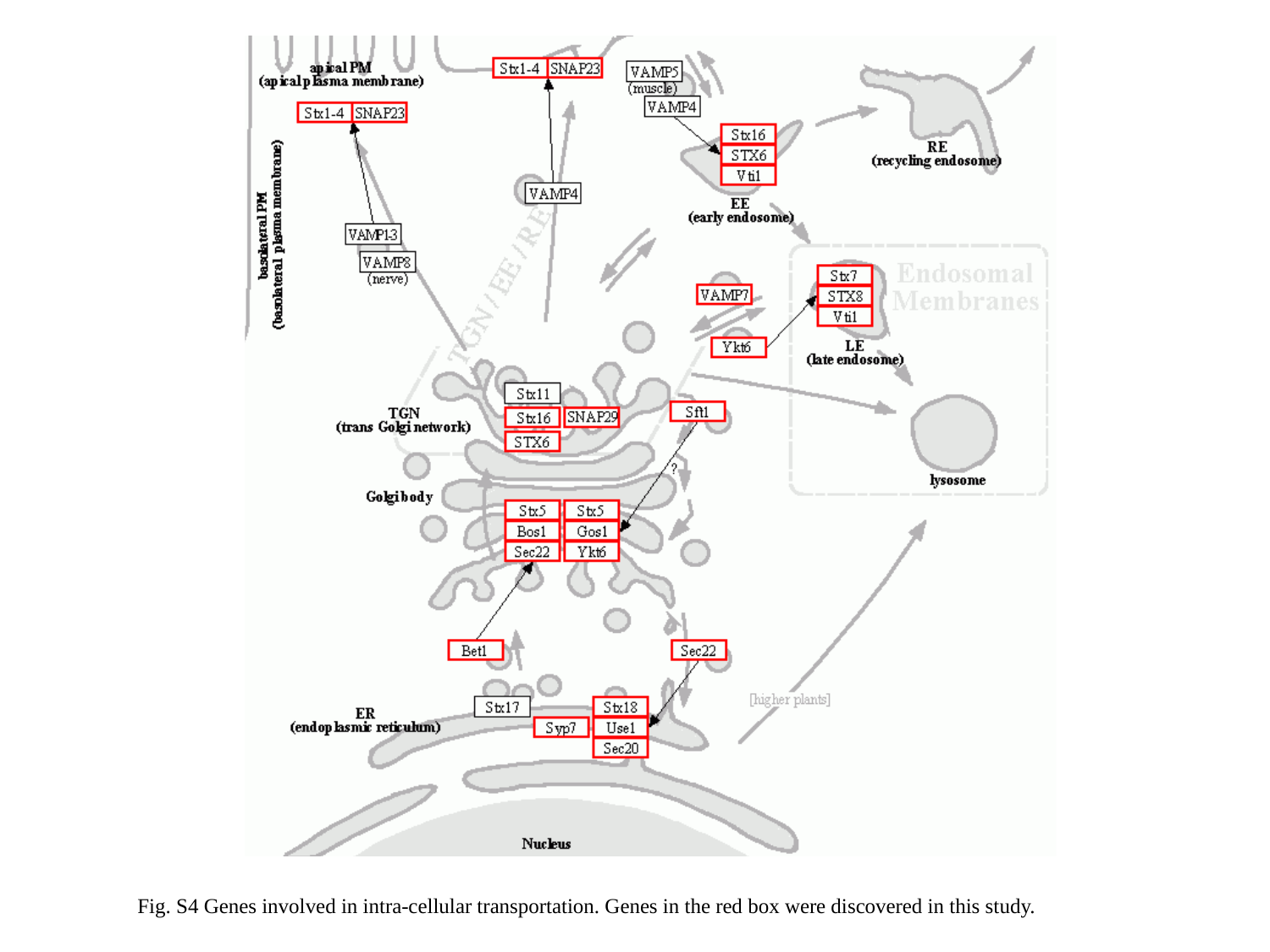

Fig. S4 Genes involved in intra-cellular transportation. Genes in the red box were discovered in this study.

## Slide 3
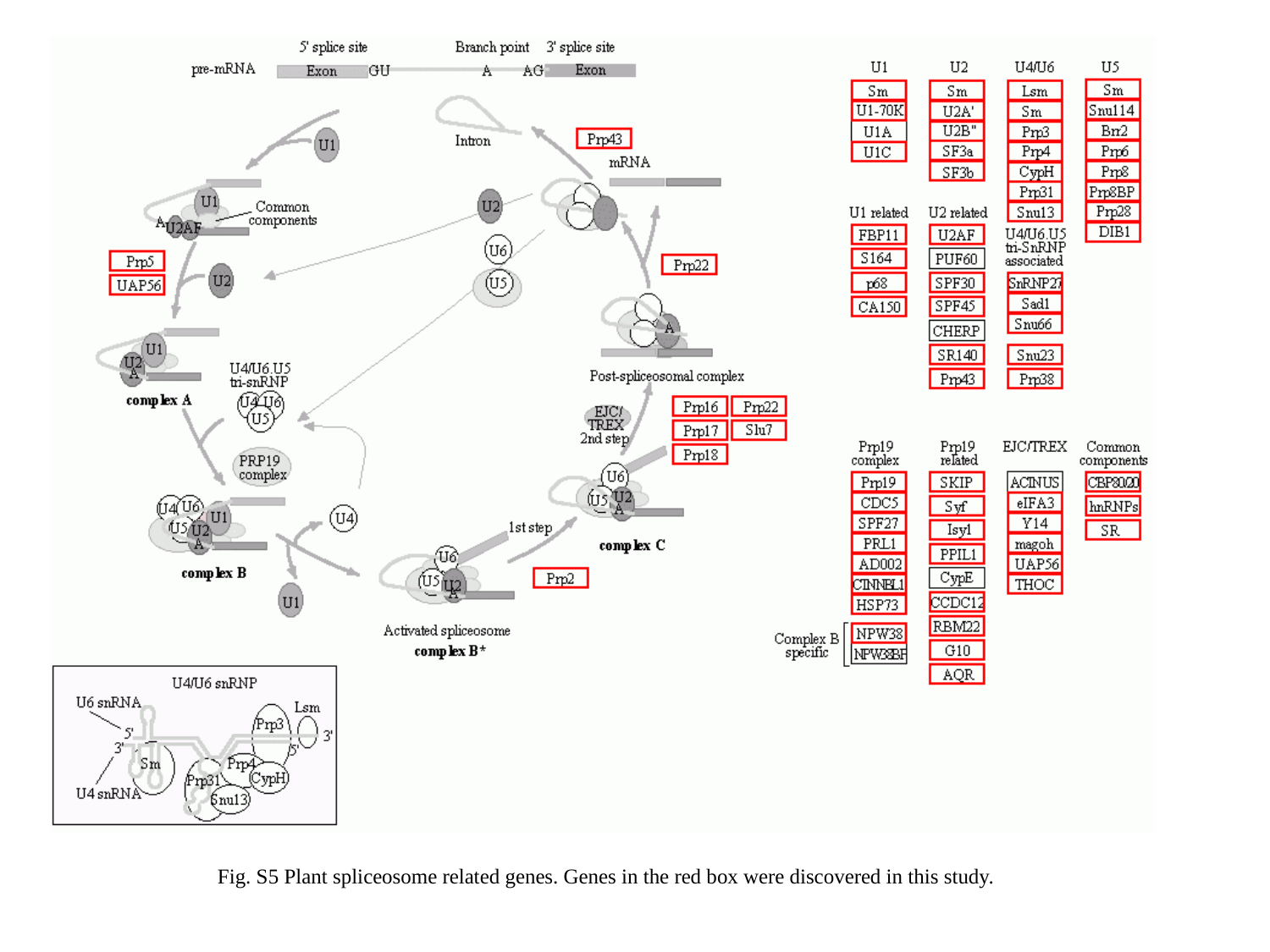

Fig. S5 Plant spliceosome related genes. Genes in the red box were discovered in this study.

## Slide 4
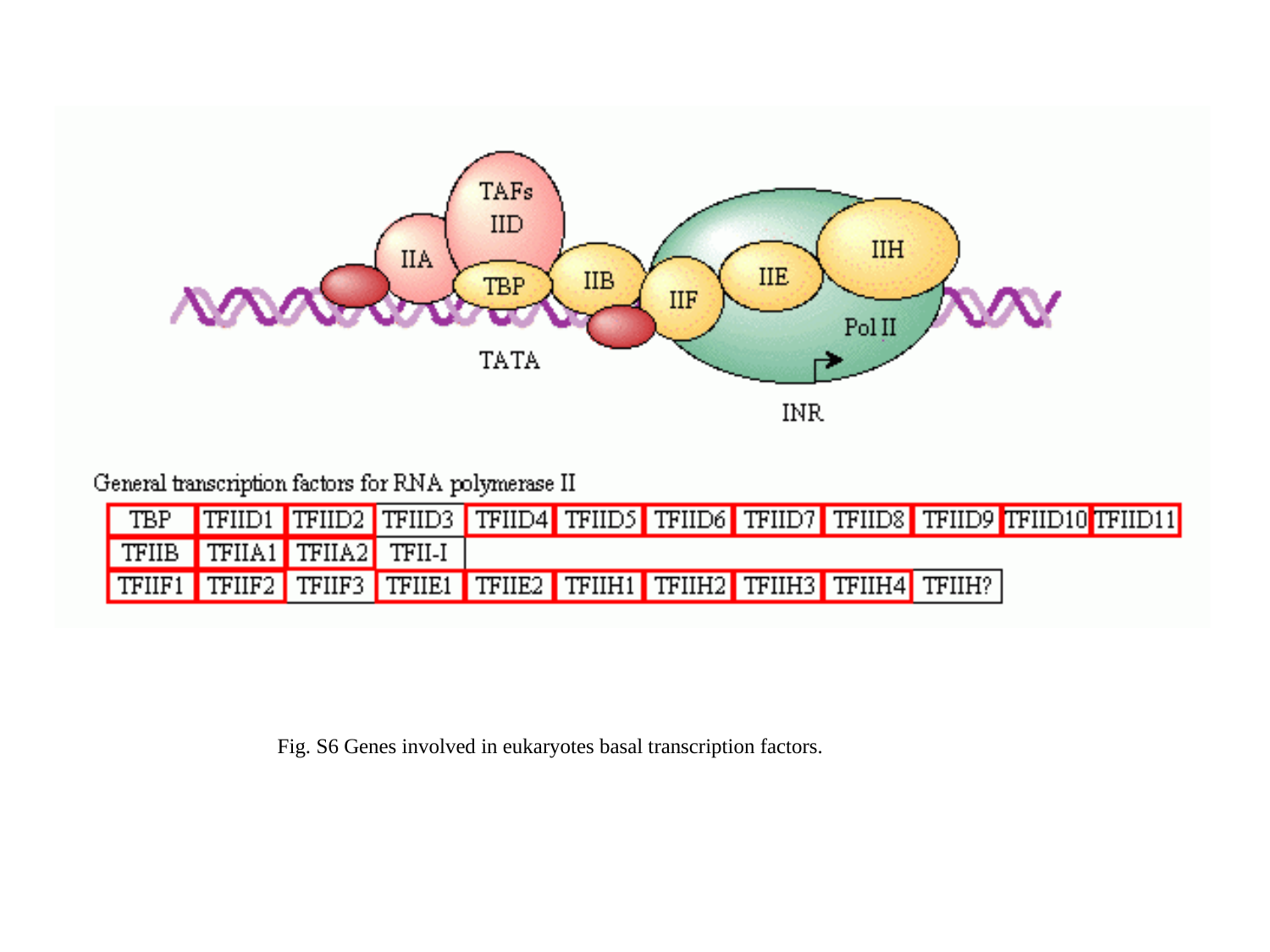

Fig. S6 Genes involved in eukaryotes basal transcription factors.

## Slide 5
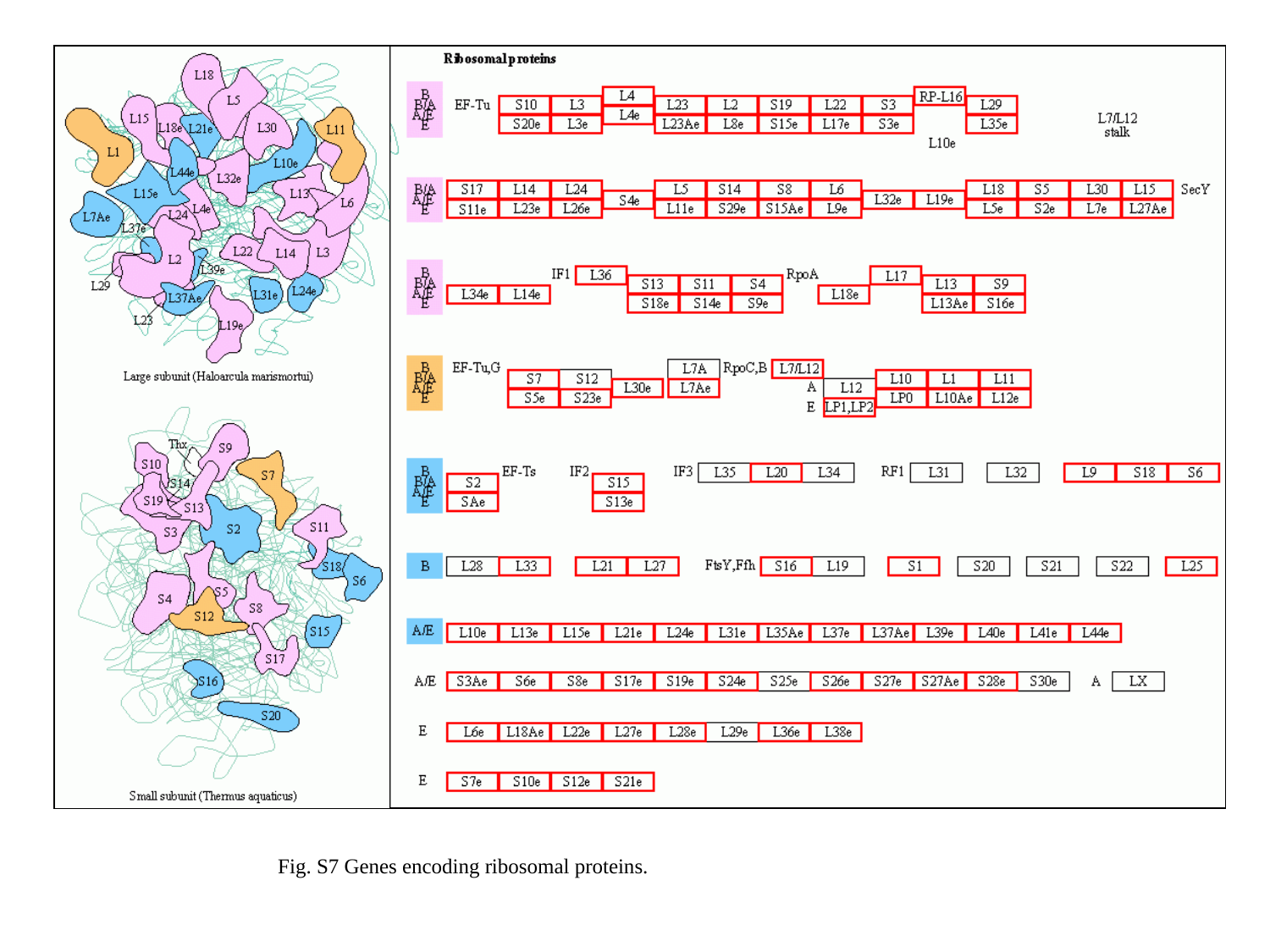

Fig. S7 Genes encoding ribosomal proteins.

## Slide 6
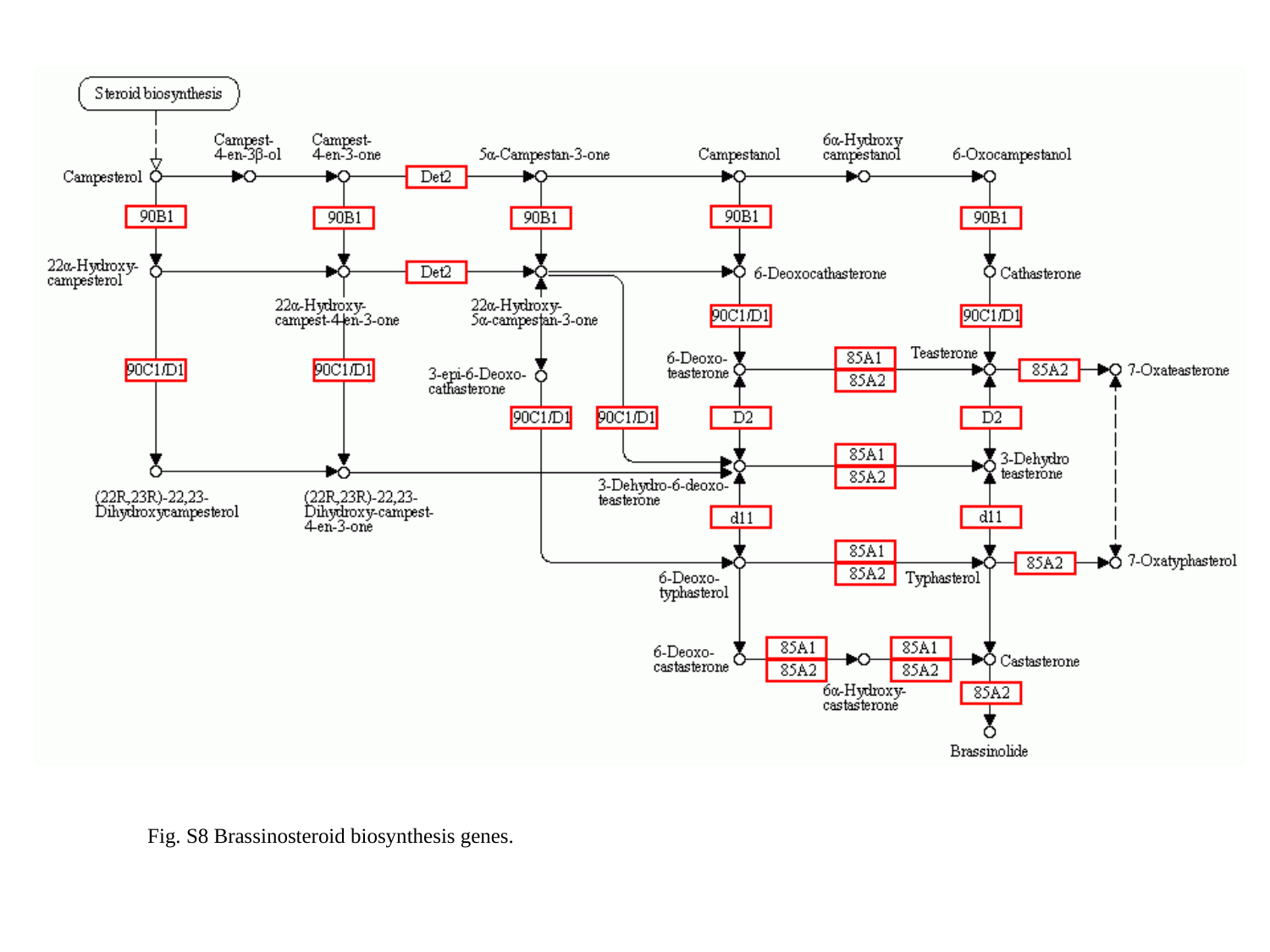

Fig. S8 Brassinosteroid biosynthesis genes.

## Slide 7
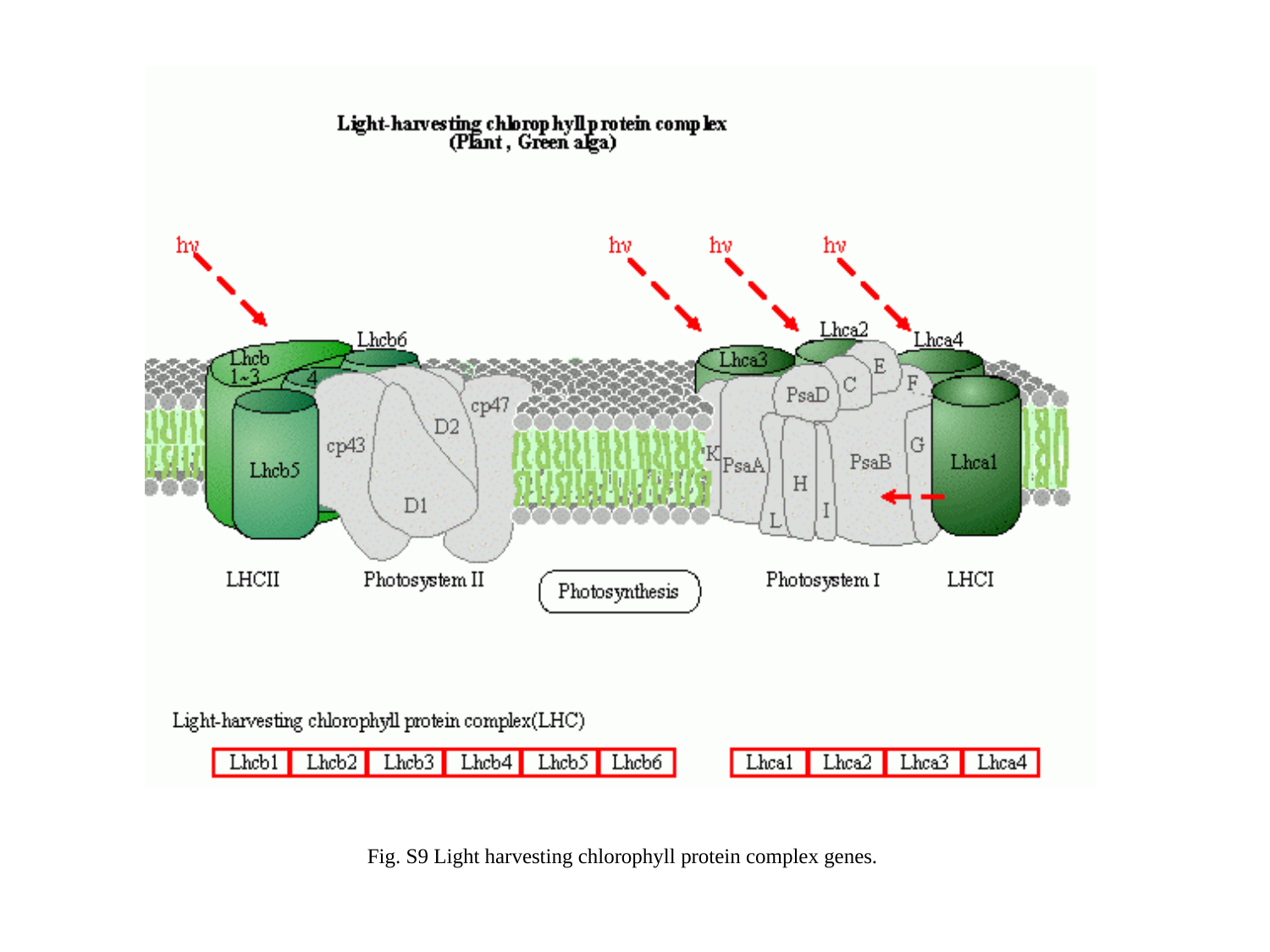

Fig. S9 Light harvesting chlorophyll protein complex genes.

## Slide 8
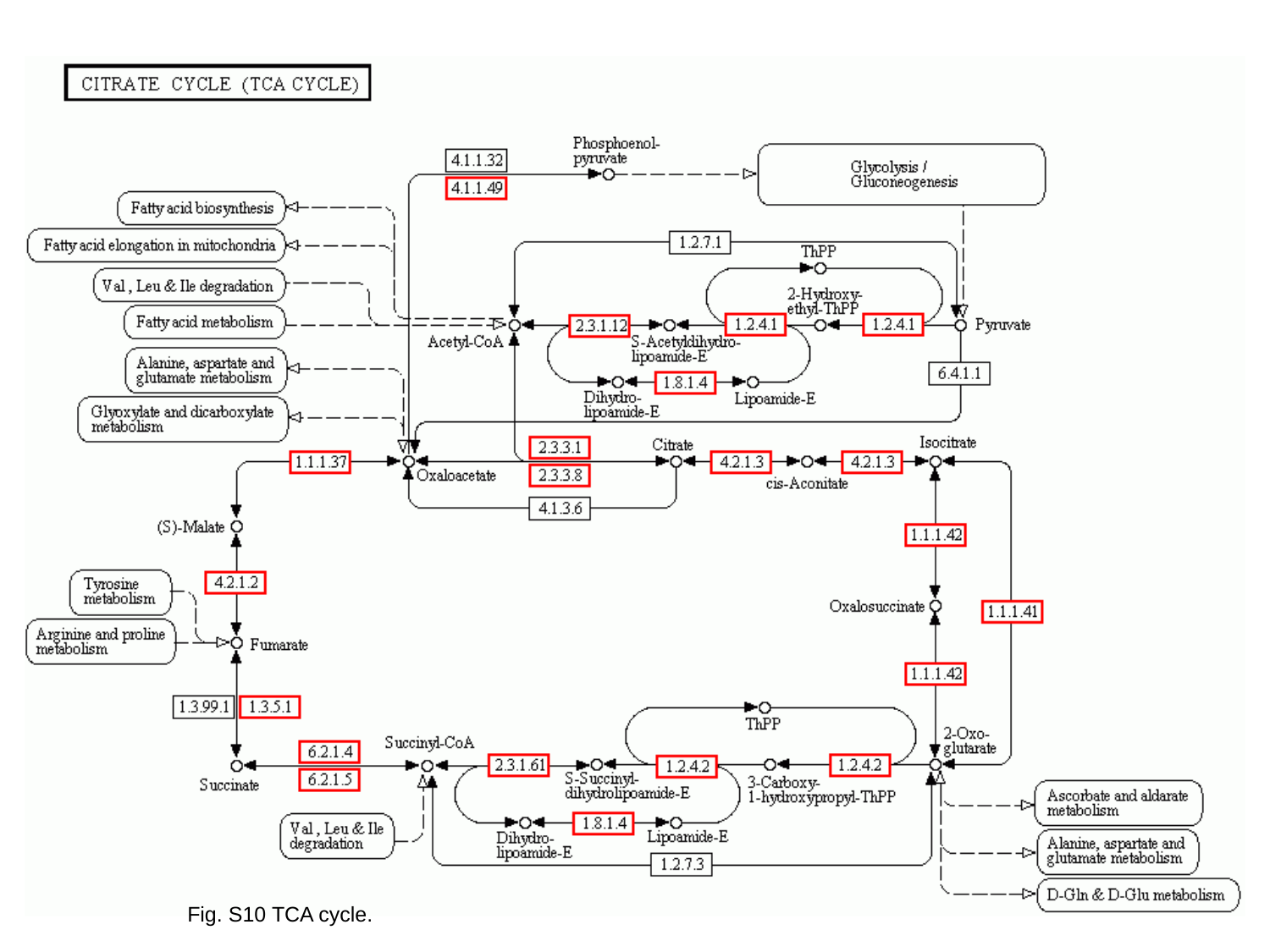

Fig. S10 TCA cycle.

## Slide 9
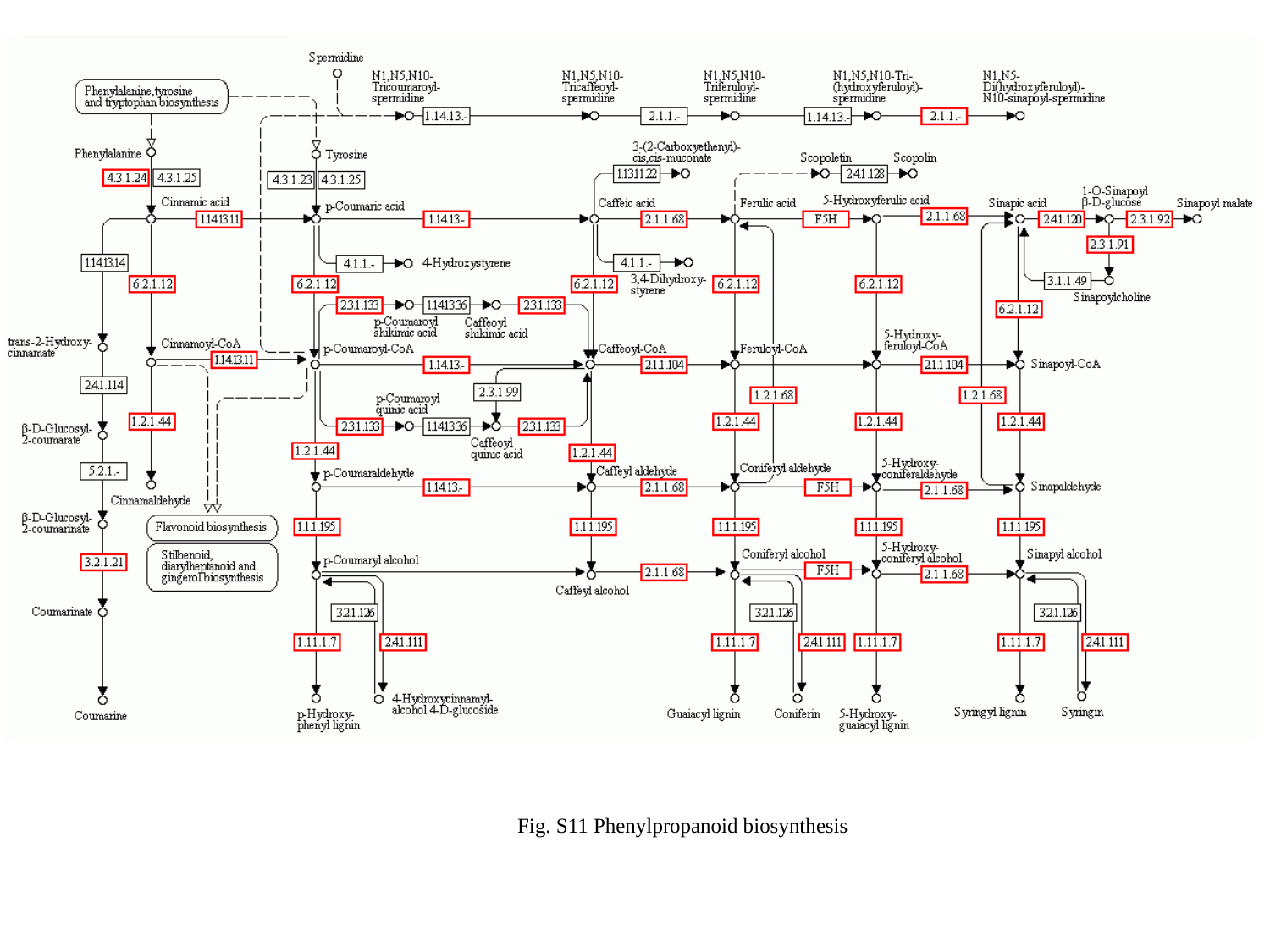

Fig. S11 Phenylpropanoid biosynthesis

## Slide 10
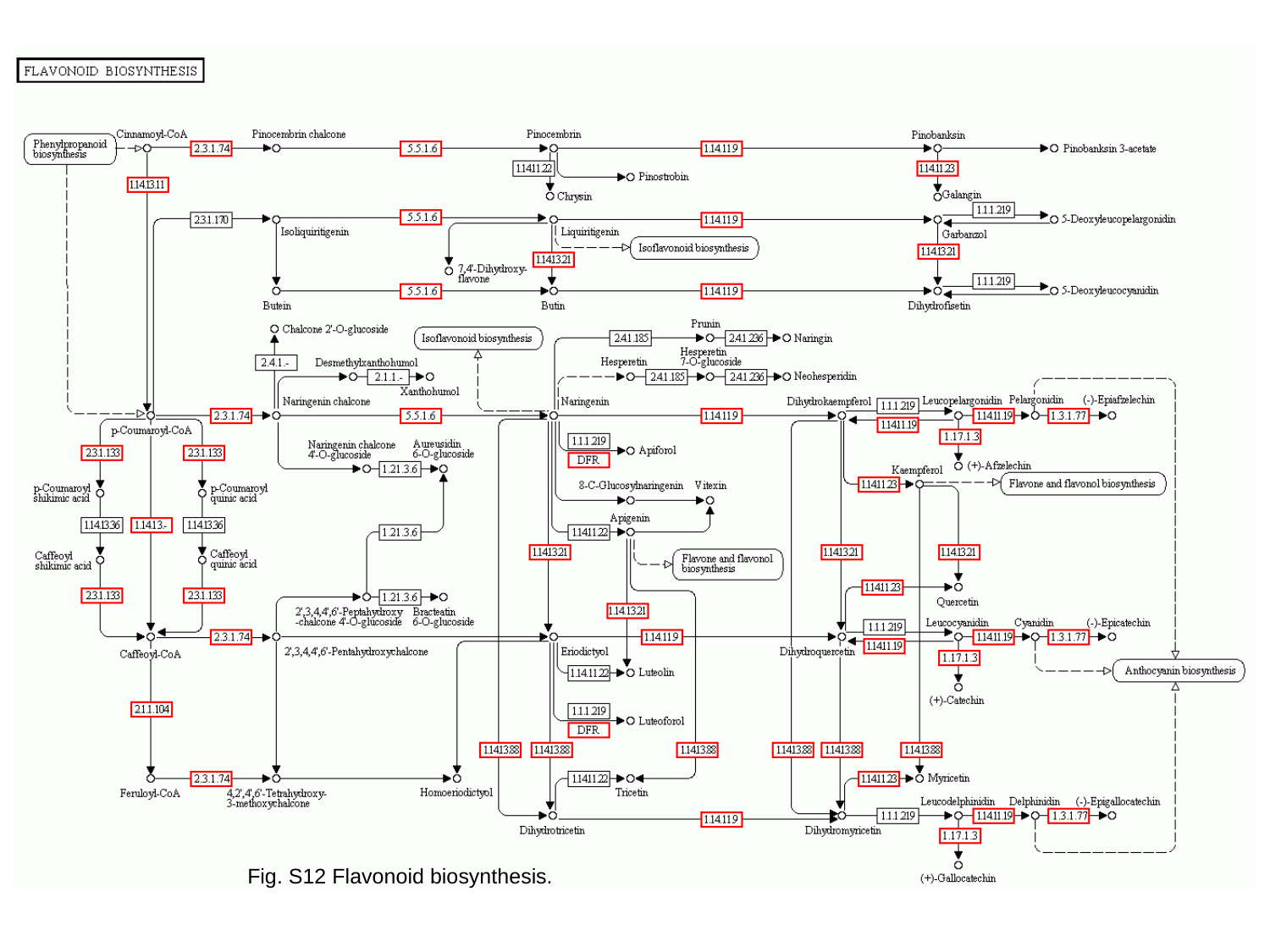

Fig. S12 Flavonoid biosynthesis.
