# Supplementary figures and images for: Transcriptome profiling of peanut gynophores revealed global reprogramming of gene expression during early pod development in darkness
Source: BMC Genomics. 2013 Jul 29;14:517. doi: 10.1186/1471-2164-14-517 (PMC3765196; doi:10.1186/1471-2164-14-517)

## Slide 1
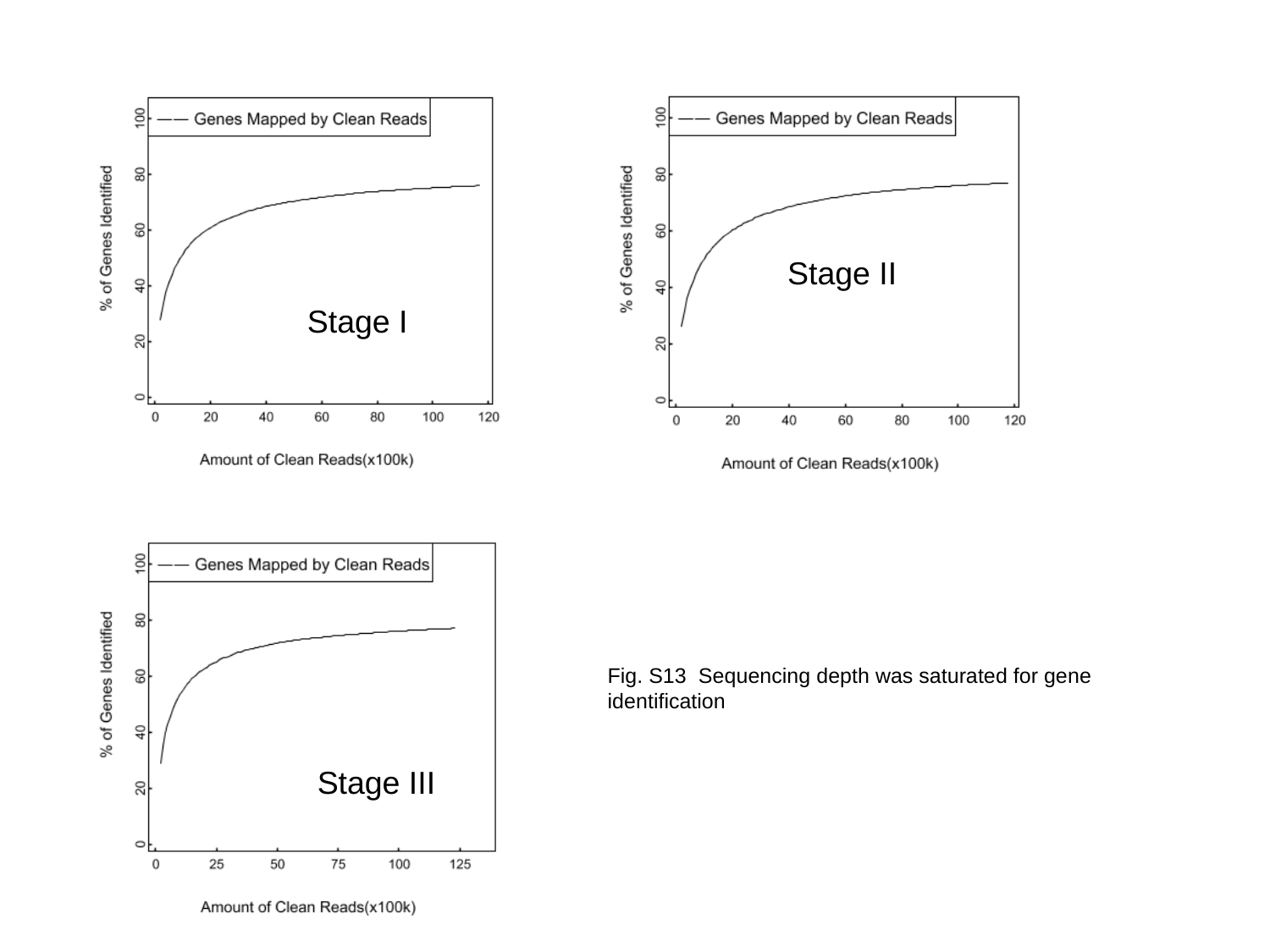

Stage I
Stage II
Stage III
Fig. S13 Sequencing depth was saturated for gene identification

Supplement: Additional file 4: Figure S13 — Sequencing depth was saturated for gene identification. S1 (stage 1), aerial grown green gynophore; S2 (stage 2), white gynophore after soil penetration without ovary enlargement; S3 (stage 3), gynophore after soil penetration and ovary enlargement. [file 1471-2164-14-517-S4.ppt]

## Slide 1
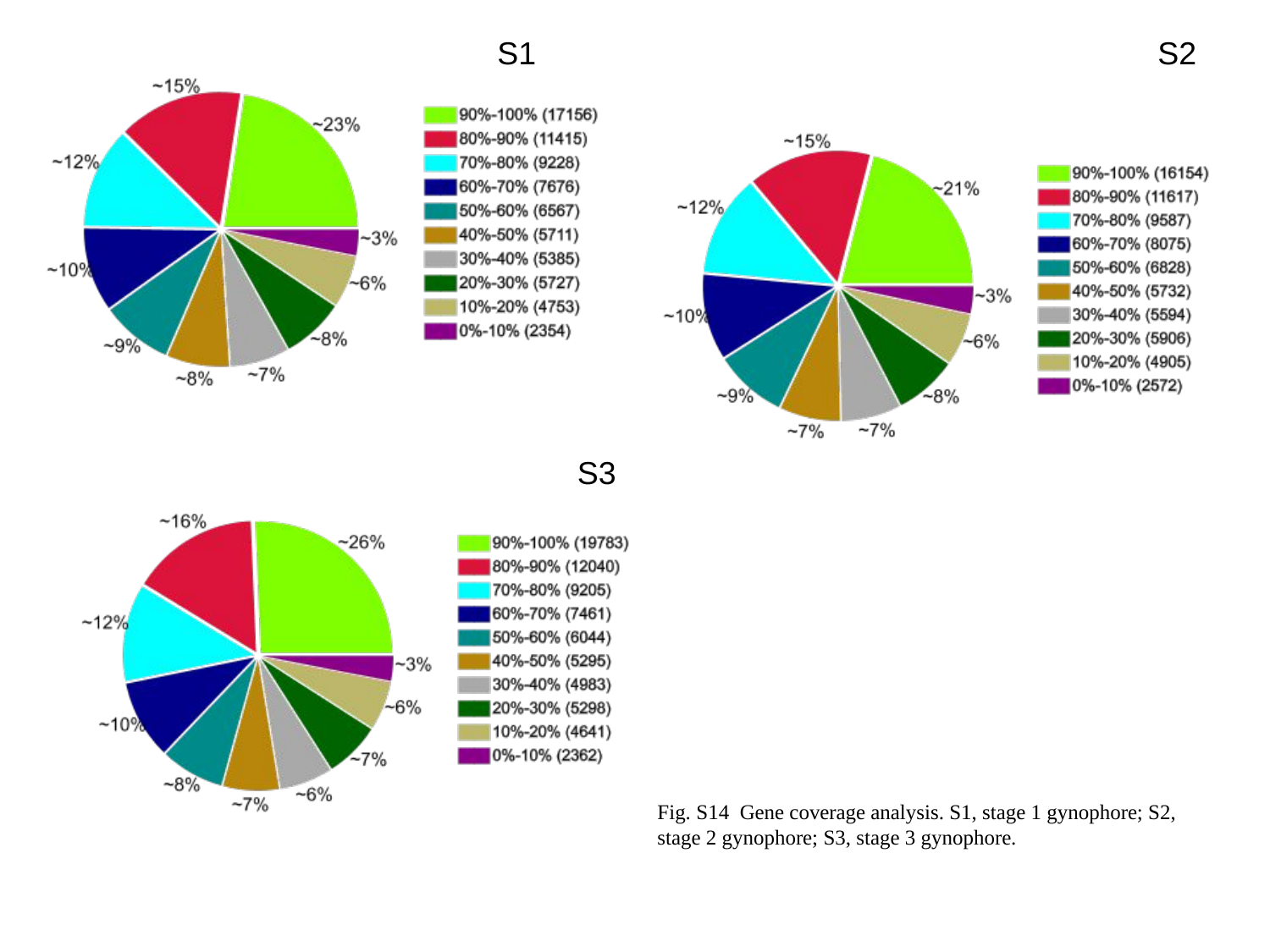

S1
S2
S3
Fig. S14 Gene coverage analysis. S1, stage 1 gynophore; S2, stage 2 gynophore; S3, stage 3 gynophore.

Supplement: Additional file 5: Figure S14 — Gene coverage analysis of S1, S2 and S3. S1 (stage 1), aerial grown green gynophore; S2 (stage 2), white gynophore after soil penetration without ovary enlargement; S3 (stage 3), gynophore after soil penetration and ovary enlargement. [file 1471-2164-14-517-S5.ppt]
